# Changing dental caries and periodontal disease patterns among a cohort of Ethiopian immigrants to Israel: 1999–2005

**DOI:** 10.1186/1471-2458-8-345

**Published:** 2008-10-02

**Authors:** Yuval Vered, Avi Zini, Alon Livny, Jonathan Mann, Harold D Sgan-Cohen

**Affiliations:** 1 Department of Community Dentistry, Hebrew University-Hadassah School of Dental Medicine, Jerusalem, Israel

## Abstract

**Background:**

Dental epidemiology has indicated that immigrants and minority ethnic groups should be regarded as high risk populations on the verge of oral health deterioration. The objectives of this study were to measure the changing pattern of dental caries, periodontal health status and tooth cleaning behaviour among a cohort of Ethiopian immigrants to Israel between the years 1999–2005.

**Methods:**

Increment of dental caries and periodontal health status was recorded among a cohort of 672 Ethiopian immigrants, utilizing the DMFT and CPI indices. Data were gathered during 1999–2000 and five years later, during 2004–2005. Participants were asked about their oral hygiene habits in Ethiopia and in Israel five years since their immigration.

**Results:**

Regarding dental caries, at baseline 70.1% of the examinees were caries-free, as compared to 57.3% after five years. DMFT had increased from 1.48 to 2.31. For periodontal health status, at baseline, 94.7% demonstrated no periodontal pockets (CPI scores 0–2) and 5.3% revealed periodontal pockets (CPI scores 3&4), compared to 75.6% and 24.4%, respectively after five years. At baseline, 74% reported cleaning their teeth exclusively utilizing chewing and cleaning sticks common in Ethiopia. After five years, 97% reported cleaning their teeth exclusively utilizing toothbrushes.

**Conclusion:**

The deterioration in the oral health status, especially the alarming and significant worsening of periodontal health status, among this immigrant group, emphasizes the need for health promotion and maintenance among immigrants and minority groups in changing societies. An "acclimatizing and integrating" model of oral health promotion among minority and immigrant groups is suggested.

## Background

Dental caries and periodontal disease have historically been considered the most important global oral health burdens [1]. International data on caries epidemiology confirm that tooth decay remains a significant disease of childhood and that the prevalence of dental caries among adults is high [1,2]. Most children and adolescents have signs of gingivitis and symptoms of periodontal disease are prevalent among adults worldwide [2]. There are profound disparities in oral health. The indigenous poor populations, immigrants, racial and ethnic minority groups, and medically compromised patients are those who suffer the worst oral health [3,4].

Recent evidence indicates that immigrants and minority ethnic groups should be regarded as "whole populations at risk" on the verge of oral health deterioration. People crossing national and cultural frontiers originally are characterized by disease patterns, health behaviours and health care modalities different from those at their destinations [5-8].

Studies conducted over the last 15 years in Israel have indicated a national decrease in caries experience, in accordance to that reported in most industrialized countries [9,10]. Fluoridation of drinking water in most large cities in Israel commenced more than twenty years ago and the use of fluoridated toothpaste is widespread. Israel is a country of immigrants from many parts of the world, including waves of immigrants from rural Ethiopia who arrived in 1984, 1991 and 1999. Ethiopia and many other African countries are characterized by poverty, scarce health education programs, lack of water fluoridation, and traditionally low sugar consumption [11,12]. Upon entering a "Westernized" society, immigrants often experience a "culture shock" that might influence their oral health status. This includes an abrupt change in nutrition and health behaviour, which for Ethiopians entails adoption of a sweeter diet and replacement of the traditional chewing and cleaning sticks common in Ethiopia with "modern" tooth brushes.

As part of an ongoing interest in and commitment to the oral health care of these populations, the Hebrew University – Hadassah School of Dental Medicine has conducted surveys which have demonstrated, among Ethiopian new immigrants, better oral health levels than those found in Israel and other "Westernized" countries [13,14].

The aim of the present study was to conduct a cohort study of Ethiopian immigrants between the years 1999–2005. The objectives were to measure the potentially changing levels of dental caries, periodontal health status and tooth cleaning behaviour between the years 1999–2005.

## Methods

During the summer of 1999 a community of about 4000 immigrants arrived from Quara, an extremely isolated rural, agricultural region of Ethiopia. Over 1000 were located in one absorption center near Jerusalem. There was no evidence to indicate any differences between the social distribution of this population and those in other centers.

The Hadassah Medical Organization, Human Experiment Ethics Committee (IRB) pre-approved this study (reference # 397-28303), in full compliance with the Helsinki Declaration . According to these guidelines, full informed consent (in Amharic) was obtained and documented before commencement of examinees' participation.

The study population included all residents of the Jerusalem center aged five years and above. Data were gathered during December 1999 to February 2000 by two calibrated examiners aided by assistants born in Ethiopia and fluent in Hebrew and Amharic. No radiographs were taken. Explanations were provided via an interpreter. Subjects were informed of any pathology found and if needed, immediately referred to the Hadassah School of Dental Medicine for first aid treatment. Dental caries status was recorded for 792 out of 795 subjects (aged >5 years), employing the DMFT index. Periodontal health status was recorded for 487 subjects (aged >12 years) employing the Community Periodontal Index (CPI). Both indices are recommended by the World Health Organization [15]. Using a portable dental chair, the examinations were conducted with the aid of a fiber optic light source and an attached dental mirror, and a special CPI probe. Subjects were asked about their oral hygiene habits in Ethiopia and in Israel since their immigration. Periodontal pockets were operationally defined as CPI scores 3 (4–5 mm) and 4 (6+ mm).

Following a government supported adaptation and acclimation period (between 6 to 18 months in the absorption center), in which efforts are invested in the process of social, cultural and financial integration, immigrants are scattered in towns across the country and expected to continue their lives as regular citizens. Social welfare services are provided on an on-going basis, where and when needed.

During the year 2004 and with the assistance of the Jerusalem absorption center management, and an Ethiopian study coordinator, 672 subjects out of the previous 792, who participated in the 1999–2000 study, and were now dispersed in communities across the country, were located and asked to participate in the cohort study. Data were gathered during July 2004 to June 2005 by one of the previous two calibrated examiners, utilizing the same methods of the 1999 examination.

Average DMFT scores were calculated for WHO recommended age groups (6, 12, 18, 35–44, 51+ years at baseline and 11, 17, 23, 40–49, 56+ years after 5 years). Differences in caries increment by independent variables (age, gender, tooth brushing at 1999–2000, tooth brushing at 2004–2005, and caries-free at 1999–2000), were univariately analyzed according to ANOVA, and multivariately (to identify independent influences), by multiple logistic regression analysis. Association of age with percentage of subjects with "worst" CPI score was tested employing the Chi-Square test. For CPI, age was grouped as 17–20, 35–44 and 51+ years at baseline and 22–25, 40–49, and 56+ years after 5 years. The Chi-square test was applied for the comparison of caries *vs*. caries-free and periodontal pockets *vs*. no periodontal pockets, for the entire population (not divided by age), over the five years. A statistical test was considered significant when p < 0.05.

## Results

The 672 respondents clinically examined in 1999–2000 (base-line examination = BL) and 2004–2005 (5 years = Y5) consisted of 315 (47%) males and 357 (53%) females. The Kappa statistic for intra and inter examiner agreement was over 90 percent and considered as adequate.

At BL, 74% of the population had reported cleaning their teeth, exclusively utilizing chewing and cleaning sticks common in Ethiopia. Five years later, 97% now reported cleaning their teeth exclusively with toothbrushes.

Mean whole population DMFT at BL was found to be 1.48 ± 3.16 and 70.1% were caries-free, as compared to 2.31 ± 4.17 and 57.3% at Y5 (p < 0.01). Mean DMFT, D, M, and F scores by age at BL and at Y5 are presented in Table 1 and differences of DMFT over time were significantly different. Mean total population caries increment score between BL and Y5 was found to be 0.90 ± 2.18 (D = 0.48, M = 0.35, F = 0.07). In all selected age groups the predominant component of the DMFT index was D (untreated caries).

**Table 1:** Distribution of caries (mean ± SD), by DMFT, D, M and F, at baseline (BL) and after 5 years (Y5), by selected age groups.

**Age (yrs)**			DMFT*		**D**		**M**		F	
**BL**	**Y5**	**N**	**BL**	**Y5**	**BL**	**Y5**	**BL**	**Y5**	**BL**	**Y5**
6	11	48	0.10 ± 0.59	0.31 ± 0.97	0.10 ± 0.59	0.24 ± 0.72	0	0.02 ± 0.23	0	0.04 ± 0.26
12	17	81	0.61 ± 1.52	0.81 ± 1.75	0.61 ± 1.52	0.75 ± 1.60	0	0.04 ± 0.44	0	0.01 ± 0.11
18	23	32	1.09 ± 1.76	1.34 ± 2.13	0.93 ± 1.62	1.00 ± 1.62	0.15 ± 0.51	0.25 ± 0.84	0	0.09 ± 0.53
35–44	40–49	85	2.41 ± 3.23	4.23 ± 4.31	1.88 ± 2.80	3.02 ± 3.18	0.51 ± 1.11	1.14 ± 1.86	0.01 ± 0.10	0.07 ± 0.25
51+	56+	42	3.71 ± 4.84	7.00 ± 6.83	1.69 ± 2.49	3.76 ± 3.57	2.00 ± 3.16	3.19 ± 4.59	0.02 ± 0.15	0.04 ± 0.30

*p < 0.001 for all selected age groups, according to two tailed t test

Among the caries-free subjects, at BL, the mean caries increment was 0.65 ± 1.75 (D = 0.48, M = 0.14, F = 0.02) as compared to a threefold higher caries increment of 1.48 ± 2.87 (D = 0.54, M = 0.76, F = 0.18) among subjects with caries at BL (p < 0.01).

In multiple logistic regressions (Table 2), the only two variables which reached statistical significance as predictors of caries increment were age and caries presence at BL. Advanced age and presence of caries at BL, increased the risk for caries increment (OR = 4.23, p < 0.01, and OR = 6.86, and p < 0.01, respectively). The combined model prediction was R^2 ^= 0.26 (p < 0.01).

**Table 2:** Multiple logistic regression model for independent variables' effect on caries increment (baseline-BL to five years-Y5)

	OR	95% CI*	p**
Age (≥ 35 yrs: ≤ 34 yrs)	4.23	2.69–6.66	0.001
Gender (males: females)	1.43	0.95–2.14	0.081
^†^Tooth cleaning at BL (non-cleaners: cleaners)	1.20	0.76–1.90	0.423
^‡^Tooth cleaning at Y5 (non-cleaners: cleaners)	2.47	0.54–11.26	0.243
Caries at BL (caries:caries-free)	6.86	4.41–10.69	0.001

^†^utilizing chewing or cleaning sticks

^‡^utilizing toothbrushes

* Confidence Interval

** two tailed statistical significance

R^2 ^for the model = 0.26 (p < 0.01)

**Figure 1:**
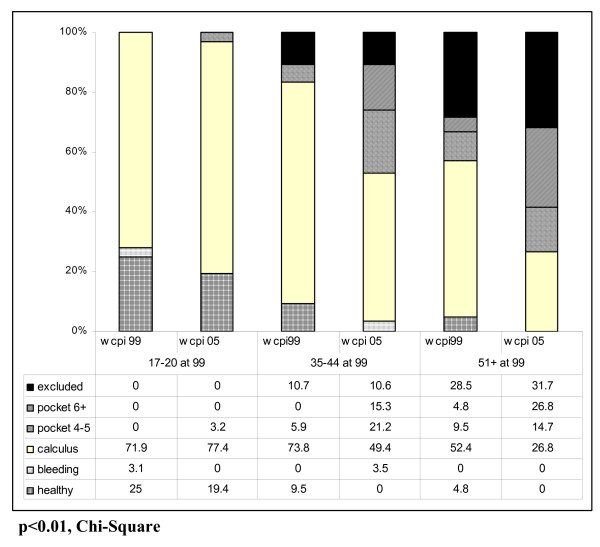
Percentage of subjects with "worst" CPI in 1999 and 2005, by age.

Three hundred and ninety one (391) respondents were clinically examined in 1999–2000 (BL >12 years) and 2004–2005 (Y5 >17 years) for periodontal status. According to CPI, 21.5% of the subjects demonstrated healthy gingival tissue at BL, as compared to only 6.2% at Y5; 64.2% had at worst, presence of bleeding or calculus at BL, as compared to 58.2% at Y5; and 5.4% exhibited periodontal pockets at BL, as compared to nearly fivefold (24.4%) at Y5. Worst CPI scores by age at BL (17–20, 35–44 and 51+ years-old) and Y5 are presented in Figure 1. Among the 35–44 and 51+ years groups, 9.5% and 4.8% of the subjects, respectively, demonstrated healthy gingival tissue at BL, as compared to no healthy subjects at Y5; 5.9% and 14.3% exhibited periodontal pockets at BL, as compared to sevenfold (36.5%) and threefold (41.5%), respectively, at Y5 (p < 0.01, Chi-Square).

**Figure 2:**
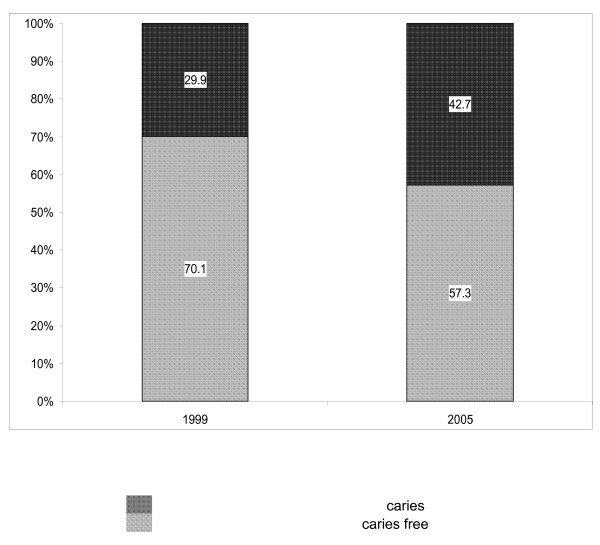
Percentage of subjects with dental health status in 1999 and 2005.

**Figure 3:**
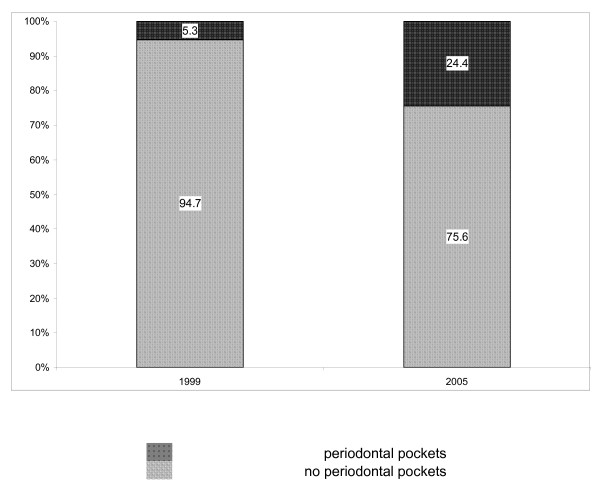
Percentage of subjects with periodontal health status in 1999 and 2005.

As demonstrated and summarized in Figure 2, among the total study participants, 29.9% had caries experience at BL, as compared with 42.7% at Y5 (p < 0.01, Chi-Square). Furthermore, as presented in Figure 3, for periodontal health status, an even more prominent deterioration was found: 5.3% had periodontal pockets at BL, as compared with about fivefold (24.4%) who had periodontal pockets at Y5 (p < 0.01, Chi-Square).

## Discussion

The present study, the first of its kind among immigrants to Israel, offers a unique opportunity to examine the changes in oral health and behaviour of a large Ethiopian immigrant cohort, upon arrival and five years later.

In a survey conducted in Israel in 1994, among new Ethiopian immigrants, mean DMFT levels of 0.31, 1.27 and 5.26 were reported among 12, 35–44 and 51+ year olds, respectively [14]. In the present study, at BL, mean DMFT levels of 0.61, 2.41 and 3.71 were found among the same age groups. These findings are in accordance with studies from Africa claiming that the previously predicted increase in caries in mainland Africa (including Ethiopia) has not occurred. In contrast to what was anticipated, caries experience is fairly stable and remains at a reasonably low level [16-18].

Notwithstanding increasing caries experience after immigration, among this Ethiopian cohort, levels remained remarkably low. Among both 6 and 12-year-olds, levels remained less than DMFT = 1, even after five years.

Among Israelis, a mean score of DMFT = 1.66 was recently found among 12-year-olds, and among 21-year- olds, a mean score of DMFT = 8.5 was demonstrated [9]. These levels can be compared with 0.81 (12 yr olds) and 1.34 (18 yr olds) in the present study.

International data have revealed that immigrants and minority ethnic groups should be regarded as prone towards oral health deterioration [5-8]. Over time, Ethiopian immigrants experience a profound change in lifestyle and culture. Following a short adaptation and acclimation period at absorption centers, the immigrants are scattered among towns across the country, as opposed to their previous rural agricultural settings in Ethiopia, and were are expected to conduct their lives as ordinary citizens.

Over a five year period, among the present total study population, caries prevalence had increased from 29.9% to 46.7% and periodontal disease (existence of periodontal pockets) prevalence had dramatically increased from 5.3% to 24.4%.

An abrupt and extreme change has occurred in the oral hygiene health behaviour of this immigrants group. At baseline, 74% reported cleaning their teeth, exclusively utilizing chewing and cleaning sticks common in Ethiopia. After five years, 97% reported cleaning their teeth, exclusively utilizing toothbrushes common in Israel and the western world. In spite of the almost unanimous report, regarding tooth brushing five years after arriving to Israel, health deterioration has occurred. The replacement of the traditional chewing and cleaning sticks common in Ethiopia by "modern" tooth brushing in Israel, might be associated with the detection of periodontal health deterioration. It should be noted that other health behaviours, especially diet, were not recorded in the present study. Based upon previous research [13], it can be assumed that a previous almost sugar-free diet had been abandoned in favour of high sugar intake.

Immigrant and minority groups in western societies require different information packages, modified strategies for forming oral hygiene habits, and encouragement to exercise discipline on factors known to be risks for oral health [5].

Population strategies are the basis for all dental public health programs, for example water fluoridation and dental health education. Although, focused relief is often necessary, models which identify and then target individuals at high risk are far from precise at individual levels [19-21]. Between these two approaches is geographic targeting, in which schools, school districts and even large regions can be identified at being at high risk [21]. Rose has discussed the advantages and disadvantages of prevention by the "whole population" strategy, and the "high-risk" strategy, and concluded that the prior concern should always be to discover and control the cause of incidence, but the two approaches are not usually in competition [20].

The present study among a large cohort of new immigrants might provide an opportunity for planning and implementing an "acclimatizing and integrating" model of oral health promotion among minority and immigrant groups, comprised of:

• Training of immigrant oral health professionals: dentists, dental hygienists, dental assistants and oral health promotion staff.

• Adaptation of an appropriate and optimal model of oral hygiene education together with consideration of previous behaviour and habits.

• Geographically targeted prevention programs in towns and neighbourhoods, populated by groups of immigrants.

## Conclusion

The combined efforts towards oral health promotion of the entire population in conjunction with an inherent commitment to its diverse subgroups should be the guiding principle for dental public health pioneers and leaders. Despite low oral disease levels (specifically for caries), even after five years, a potential future disease increase has been indicated. Public health attention should therefore not be averted from the potential hazards of changing societies on the health status of migrant populations.

## Competing interests

The authors declare that they have no competing interests.

## Authors' contributions

YV conducted the examinations and participated in the manuscript presentation, AZ contributed to the statistical analyses, AL participated in the data analysis and presentation, JM and HSC participated in the design and presentation of the study.

## Pre-publication history

The pre-publication history for this paper can be accessed here:


